# Simultaneous Determination of Twenty-Five Compounds in Rat Plasma Using Ultra-High Performance Liquid Chromatography-Polarity Switching Tandem Mass Spectrometry and Its Application to a Pharmacokinetic Study

**DOI:** 10.3390/molecules22111853

**Published:** 2017-10-29

**Authors:** Na Zhang, Yueting Li, Jing Sun, Chun Li, Yuelin Song, Jun Li, Pengfei Tu, Yunfang Zhao

**Affiliations:** 1 Modern Research Center for Traditional Chinese Medicine, School of Chinese Materia Medica, Beijing University of Chinese Medicine, Beijing 100029, China; zhangna5677@163.com (N.Z.); yuetingli1111@163.com (Y.L.); ycsunjing2008@126.com (J.S.); lichun19850204@163.com (C.L.); syltwc2005@163.com (Y.S.); drlj666@163.com (J.L.); pengfeitu@bjmu.edu.cn (P.T.); 2 Department of pharmacy, Baotou Medical College, Baotou 014060, China

**Keywords:** *Qishen Keli*, multi-component pharmacokinetics, polarity-switching multiple reaction monitoring, simultaneous determination, ultra-high performance liquid chromatography

## Abstract

An attempt was made to characterize the pharmacokinetic profiles of *Qishen Keli* (QSKL) that has been widely proved to be effective in clinical practice. A method using ultra-high performance liquid chromatography coupled with tandem mass spectrometry (UHPLC-MS/MS) for the simultaneous determination of 25 analytes in rat plasma was developed and validated. Satisfactory chromatographic separation was achieved on an ACQUITY UPLC HSS T3 column with gradient elution using mobile phase consisting of 0.02% aqueous formic acid (A) and acetonitrile fortified with 0.02% formic acid (B), and analyte detection was carried out using polarity-switching multiple reaction monitoring mode. Method validation assays in terms of selectivity, linearity, inter- and intra-day variations, matrix effect, and recovery demonstrated the newly developed method to be specific, sensitive, accurate, and precise. Following the oral administration of QSKL at a single dose, the qualified method was successfully applied for pharmacokinetic investigations in sham and model rats. Mild differences occurred for the pharmacokinetic patterns of most components between those two groups, whereas significant differences were observed for glycyrrhizic acid and glycyrrhetic acid. The obtained findings could provide meaningful information for the clarification of the effective material basis of QSKL.

## 1. Introduction

Traditional Chinese medicines (TCMs) are playing an increasingly crucial role for the treatment of various chronic disorders, because they are multi-component and multi-target agents, thus leading to a holistically therapeutic action for multi-factorial diseases [1,2,3,4,5]. The efficacy of TCMs has been well-defined by more and more evidences from clinical practices; however, it is still challenging to clarify the effective material basis of a given TCM. Because exposureto a component in the circulation system is the prerequisite for efficacy in most cases, it is thereby viable to characterize the effective constituents via profiling the compounds and assessing their exposure patterns in plasma. Moreover, different pharmacokinetic profiles can usually observed between normal and pathological subjects, attributing to their different physiological status; hence, pharmacokinetic comparisons should provide direct clues for the characterization of therapeutic components. In the other words, multi-component pharmacokinetic evaluation [6,7,8,9], especially comparison between normal and diseased groups, is a feasible approach to find the ingredients being responsible for TCM efficacy.

*Qishen Keli* (QSKL) is derived from two well-known TCM formulas, namely *Simiaoyongan* and *Zhenwu* decoctions, and consists of six famous herbal medicines: Astragali Radix (Chinese name: Huangqi), Salvia Miltiorrhiza Radix et Rhizoma (Chinese name: Danshen), Lonicerae Japonicae Flos (Chinese name: Jinyinhua), Scrophulariae Radix (Chinese name: Xuanshen), Aconiti Laterlis Radix Preparata (Chinese name: Fuzi), and Glycyrrhizae Radix et Rhizoma (Chinese name: Gancao). It has been widely used in the clinic by a number of notable physicians for the treatment of coronary heart disease patients with cardiac insufficiency [10]. In our previous studies, rats with myocardial ischemia simulated by left anterior descending coronary artery ligation havebeen proved to be a reliable heart failure model, and a set of investigations have also been carried out concerning the underlying pharmacological mechanisms for the therapeutic benefits of QSKL in pathological rats [11,12,13,14,15]. The primary signal pathways involved in the therapeutic actions have been revealed as well [11,12,13,14,15], however, the components responsible for the therapeutic outcomes remain unclear. Therefore, an attempt to reveal the effective components, the current study through a multi-component pharmacokinetic study of QSKL in both sham and model rats was carried out.

Ultra-high performance liquid chromatography-tandem mass spectrometry (UHPLC-MS/MS) integrates the superior separation potential of UHPLC along with the excellent specific and sensitive detection of MS/MS; hence, it is unsurprising that UHPLC-MS/MS can serveas a workhorse for the simultaneous determination of dozens of analytes in complicated matrices, e.g., plasma [16], feces [17,18], urine [19], etc. In general, a large array of components can be generated in vivo following oral administration of a given TCM. Because of the challenges regarding chromatographic separation as well as mass spectrometric detection, only several primary components were selected as analytes in numerous previous studies [20,21,22] although they aren’t able to holistically reflect the pharmacokinetic properties of the TCMs. Fortunately, some new bonding and end capping technologies, such as a tri-functional C_18_ alkyl phase bonding at a ligand density and polar end capping, are recently drawing worldwide interest attributing to the significant improvements in regard of polarity range, pH span, lifespan, and peak shape have been achieved, in comparison of those conventional RP-C_18_ columns [23,24,25,26], suggesting a promising tool for separating tens of components. On the other side, some of those xenobiotics may prefer positive ionization polarity, while others are better analyzed with negative polarity. In practice more than 500 milliseconds are usually required for the transformation between different ionization polarities, although some innovative techniques have been applied to the ion sources of mass spectrometers. A typical peak from UHPLC usually exhibits a width amongst 10–20 s. Generally speaking, more than 12 data-points are mandatory for reproducible quantification, and it is thereby challenging to acquire enough data-points for each peak when polarity-switching always occurs within a single analytical run. As a work-around, analysts usually measure a single sample applying positive and negative polarities in two separate runs, which represents a significant barrier for high-throughput assay [27,28]. Fortunately, a more efficient approach, that is fragmenting an entire measurement into several periods is permitted by modern mass spectrometers. Therefore, a single polarity is implemented in one period and polarity conversion is only scheduled between adjacent periods [29].

**Figure 1:**
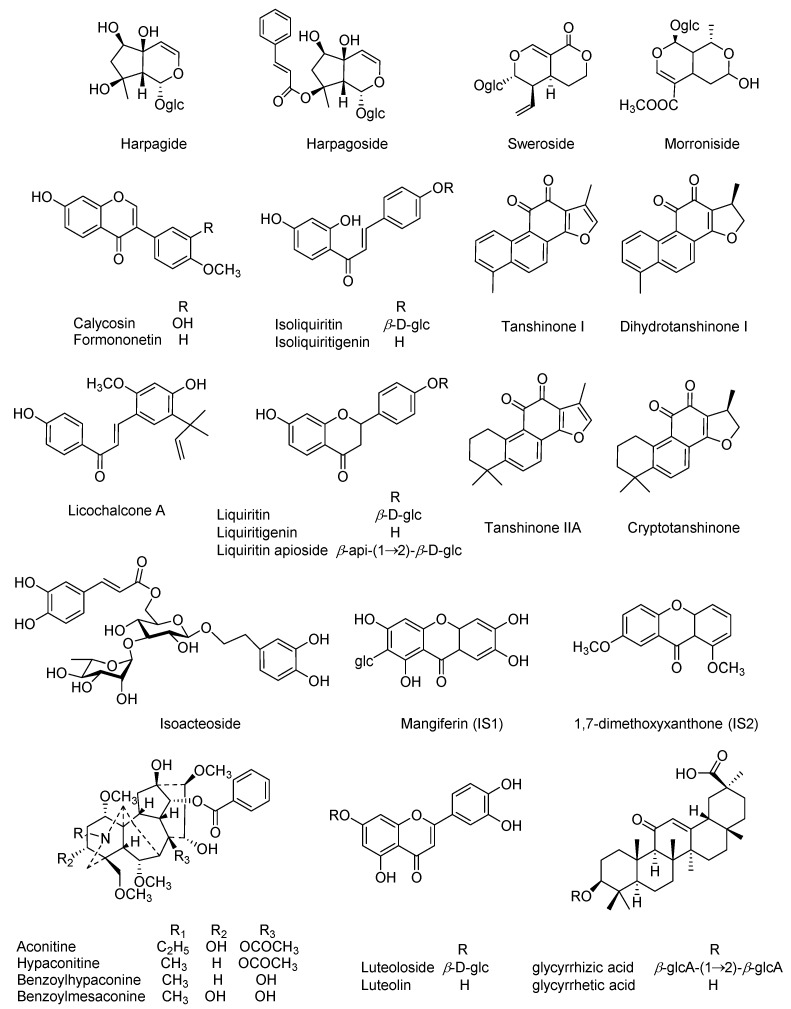
Chemical structures of the twenty-five analytes and internal standards.

Our preliminary experiments have characterized the prototypes as well as the metabolites in rat plasma following oral administration of QSKL, and twenty-five compounds (Figure 1) were observed as the primary QSKL-derived components. Herein, simultaneous determination of those compounds in rat plasma was attempted using UHPLC-polarity switching MS/MS (UHPLC-*ps*MS/MS), and the developed method was applied to characterize their kinetic patterns in both sham rats and myocardial ischemia rats. We envisioned that the obtained findings could offer a practical approach for multi-component pharmacokinetic evaluations of TCMs, while providing solid information for the clarification of the effective material basis of QSKL.

## 2. Results and Discussion

### 2.1.Method Development

It is very difficult to achieve satisfactory chromatographic profiles for TCM-treated plasma, because the complicated chemical pool contains both the components coming from the TCM and the endogenous substances found in plasma. Hence, several columns that were claimed to be robust were screened here to accomplish an acceptable separation for those twenty-five analytes, particularly between those compounds with different ionization polarities. A Waters ACQUITY UPLC HSS T3 column (100 mm × 2.1 mm, 1.8 μm, Waters, Wexford, Ireland) was ultimately employed because it offered advantages of both peak capacity and peak shape, in comparison with Kinetex-C_18_ core-shell column (100 mm × 2.1 mm, 2.6 μm, Phenomenex, Torrance, CA, USA), ACE UltraCore 2.5 SuperC_18_ column (150 mm × 3.0 mm, 2.5 μm, Advance Chromatography Technologies Ltd., Aberdeen, UK), and Waters ACQUITY UPLC BEH column (100 mm × 2.1 mm, 1.7 μm), as well as the Capcell core ADME column (150 mm × 2.1 mm, 2.7 μm, Shisheido, Tokyo, Japan), because the T3 column utilizes attractive technologies in terms of bonding and end-capping, and it was thereby able to afford satisfactory chromatographic behaviors for all compounds of interest. 

Regarding manual mass parameter tuning, each analyte stock solution was diluted to an appropriate concentration (50–100 ng/mL) with 50% aqueous methanol, and directly infused into the ESI interface at a flow rate of 7 μL/min using a syringe pump. The investigated analytes along with the internal standards were firstly measured to yield both MS^1^ and MS^2^ spectra to ascertain their precursor-to-product ion transitions for quantitative analysis. The positive and negative ionization modes were compared, and the results proved that the positive mode could provide higher responses for some analytes, such as benzoylmesaconine, benzoylhypaconine, aconitine, hypaconitine, dihydrotanshinone I, cryptotanshinone, tanshinone I and tanshinone IIA, and IS2, whereas the other analytes including harpagide, morroniside, sweroside, liquiritin apioside, liquiritin, luteoloside, isoacteoside, isoliquiritin, harpagoside, liquiritigenin, luteolin, calycosin, isoliquiritigenin, glycyrrhizic acid, formononetin, licochalcone A, and glycyrrhetic acid, and IS1 were found to be more suitable for negative polarity. After careful manual optimization, two precursor-to-product ion transitions, including quantifier and qualifier ones, were set for each analyte, and the more sensitive one (Table 1) served as the quantifier one. Ion source gas flows and ion source temperature adopted the typical values corresponding to the UHPLC effluent of 0.4 mL/min.

Moreover, mobile phase modifiers were screened among ammonium acetate, formic acid, acetic acid, and ammonium formate by comparing the overall response of allanalytes. Formic acid was finally introduced as the modifiers for both organic (solvent B) and aqueous (solvent A) solvents to strengthen the ionization of most analytes. Either contentof formic acid was optimized as 0.02% (*v*/*v*). The optimized chromatography and mass spectrometry parameters are discussed in Section 4.2 and listed in Table 1.

### 2.2. Method Validation

#### 2.2.1. Selectivity and Specificity

**Figure 2:**
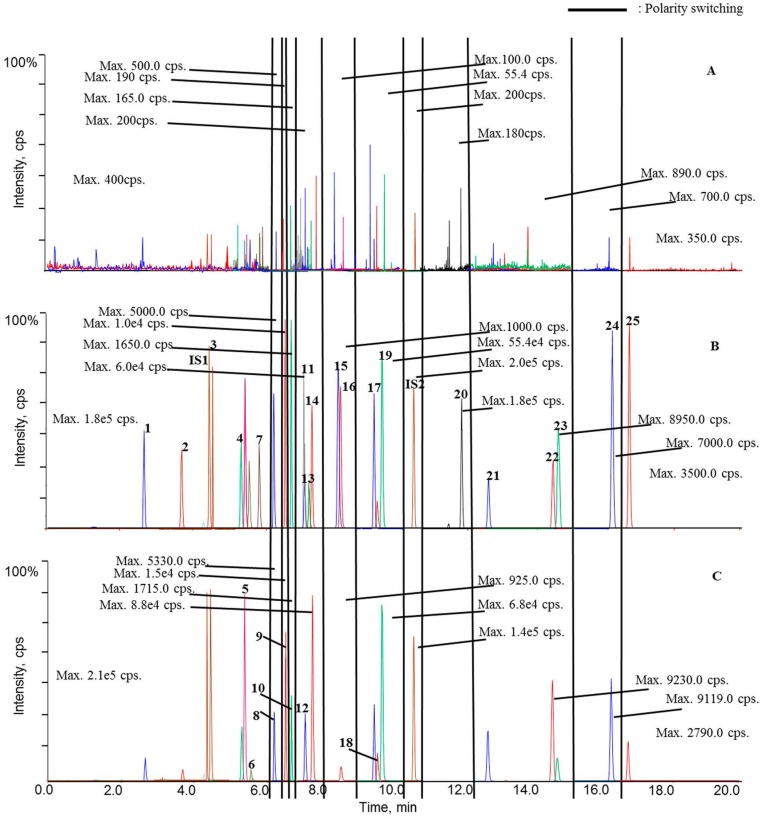
Typical chromatograms of blank plasma (**A**); blank plasma sample spiked with twenty-five analytes and internal standards (**B**); and plasma sample 1.0 h from a selected sham rat after oral administration of QSKL (**C**). As described above, polarity switching (marked with black lines) occurs for eleven times. Each segment is appropriately magnified to assure every signal visible, and the intensities of all the base peaks (highest signals) are shown. (**1**) harpagide; (**2**) morroniside; (**3**) sweroside; (**4**) liquiritin apioside; (**5**) liquiritin; (**6**) luteoloside; (**7**) isoacteoside; (**8**) benzoylmesaconine; (**9**) isoliquiritin; (**10**) benzoylhypaconine; (**11**) harpagoside; (**12**) liquiritigenin; (**13**) luteolin; (**14**) calycosin; (**15**) aconitine; (**16**) hypaconitine; (**17**) isoliquiritigenin; (**18**) glycyrrhizic acid; (**19**) formononetin; (**20**) licochalcone A; (**21**) dihydrotanshinone I; (**22**) cryptotanshinone; (**23**) tanshinone I; (**24**) glycyrrhetic acid; (**25**) tanshinone II_A_.

Retention times of the twenty-five analytes, IS1 and IS2 are summarized in Table 1, while Figure 2 displays the representative chromatograms of calibration samplesas well asplasma samples obtained from sham rat at 0 h (blank plasma) and 1 h after oral administration. Overall, satisfactory chromatographic performance was achieved for these analytes using the current UHPLC-*ps*MS/MS program (Figure 2B). 

The signals were assigned to analytes and internal standards by comparing retention times, response ratios between the quantifier and qualifier ion transitions, and MS^2^ spectra with those of authentic compounds. Although minor responses (lower than 100 cps for each) were observed for some endogenous substances around the signals of certain analytes, those weak signals would not significantly affect the reliable quantitation of those analytes.

#### 2.2.2. Linearity, LOQ and LOD

Regressive linear equations, correlation coefficients, linear ranges, LOQs, and LODs of the twenty-five analytes are summarized in Table 2. All calibration curves showed good linearity within individually concentration ranges with correlation coefficients (*r*) ranged from 0.9978 to 0.9995. LOQs and LODs of analytes varied amongst the ranges 7.11 pg/mL–6.60 ng/mL and 0.900 pg/mL–1.89 ng/mL, respectively. Above all, the linear properties along with sensitive features could fulfill the requirements of the multi-component pharmacokinetic studies.

#### 2.2.3. Precision and Accuracy

Inter- and intra-day precisions of the method for the simultaneous determination of twenty-five analytes were studied using QC samples, and the RSD% values, which ranged from 1.9% to 15.5%, are presented in Table 3. REs of all concentration levels in the linearity range of all analytes were between 85.3–115.0%, indicating that the accuracy could also meet the requirements of the pertinent guidelines. Those findings proved the newly developed method to be precise and accurate.

#### 2.2.4. Matrix Effect and Recovery

The overall method recovery was governed by two terms such as matrix effect and recovery, and the overall method recovery played a pivotal role for the assessment of the method validity. Matrix effect and recovery assays were utilized to evaluate the interferences for the targeted components from inherent substances in the matrices and the influence of extraction process, respectively. Following the preparation of those three sets (sets A–C) of samples, comparisons were performed among their quantitative results. The overall method recoveries were calculated among 58.4–91.8% (RSD% among 2.6–16.3%), while the recovery (Table 4) and the matrix effect (Table 5) ranged from 61.3% to 90.4% (RSD% among 1.3–13.9%) and from 91.6% to 107.0% (RSD% among 1.0–10.1%), respectively. Because 25% of the plasma sample was deserted to completely remove those precipitates during sample preparation, the recovery as well as the overall method was approximately 75%. Given the parallel sample preparation between calibration samples and real samples, the relative low recovery could not affect the acquisition of reliable results. Above all, the overall method recovery could not obstruct reliable quantitation.

#### 2.2.5. Stability

Stability of the twenty-five analytes under conditions of room temperature for 24 h, three freeze-thaw cycles, and −80 °C for 30 days were assayed. The results are summarized in Table 6, and it showed the deviations (RE) between measured values and nominal values were among 81.6–117.0%, the RSD% values ranged from 0.5% to 15.9%. Therefore, all analytes kept intact in all stability assays.

### 2.3. Application in Pharmacokinetic Study

As aforementioned, our preliminarystudy disclosed that twenty-five ingredients in QSKL could be detectable in the circulation system following oral administration; hence, these components were employed as markers to reflect the pharmacokinetic pattern of the entire QSKL formula. The validated method was applied for pharmacokinetic investigations of QSKL in the sham and model rats. Among thetwenty-five analytes, trace distributions were observed for four compounds, such as isoacteoside, luteolin, aconitine, and licochalcone A, and these four compoundsofinterestwere unquantifiable in most QSKL-treated samples even that the dosage was set at 8.0 g/kg (Figure 2C). Meanwhile, significant distributions were detected for the other twenty analytes, including harpagide, morroniside, sweroside, liquiritin apioside, liquiritin, luteoloside, benzoylmesaconine, isoliquiritin, benzoylhypaconine, liquiritigenin, calycosin, hypaconitine, isoliquiritigenin, glycyrrhizic acid, formononetin, dihydrotanshinone I, cryptotanshinone, tanshinone I, glycyrrhetic acid, and tanshinone IIA, in rat plasma after a single oral dose of QSKL because they were quantifiable in most samples (Figure 2C).

**Figure 3:**
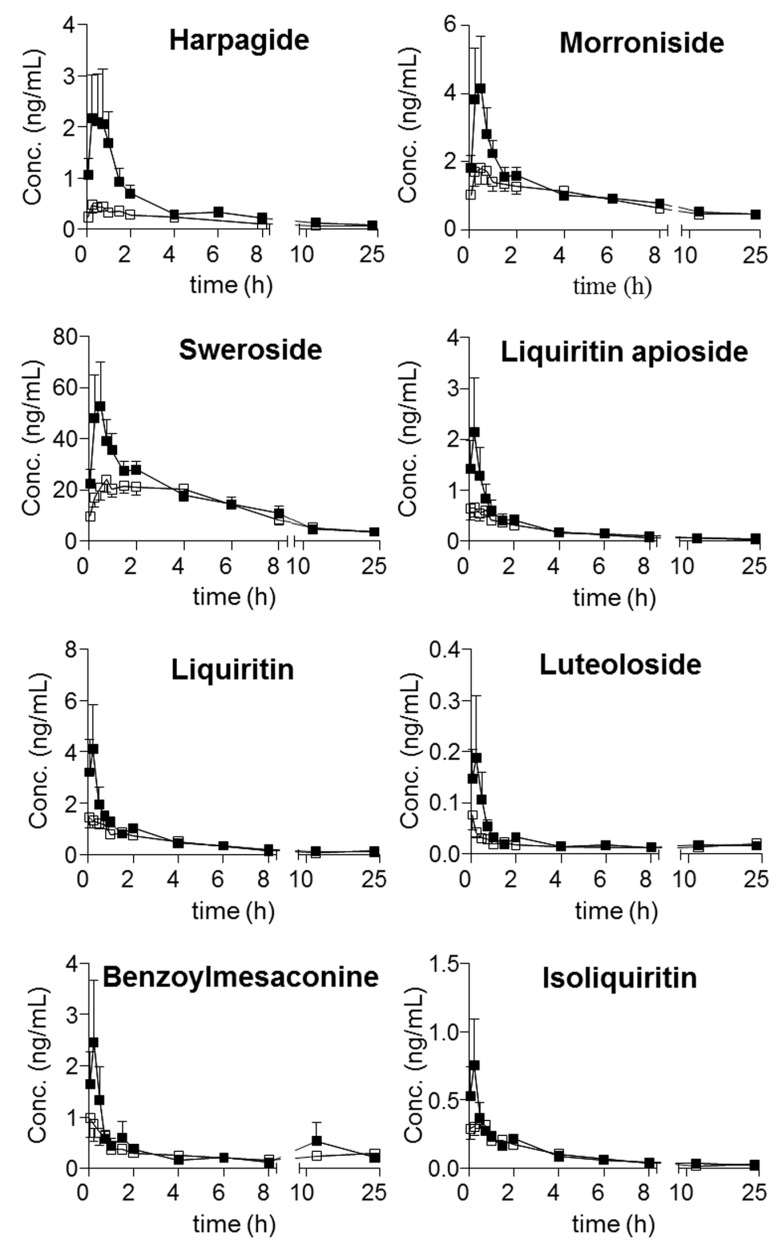

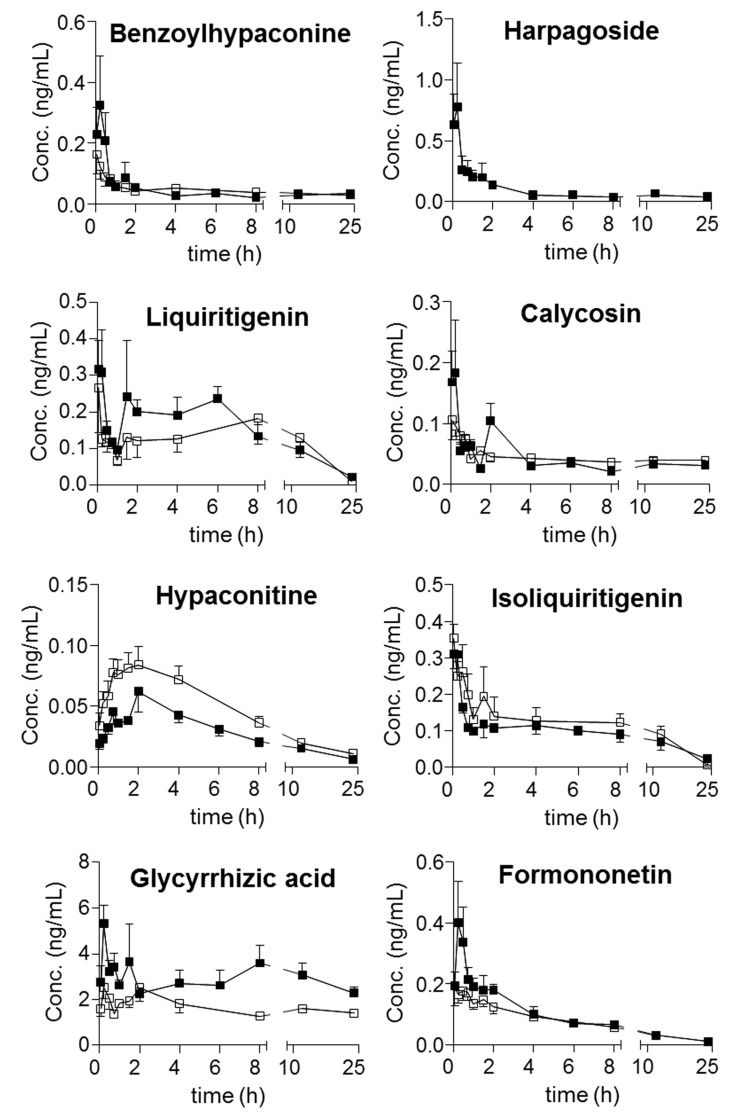

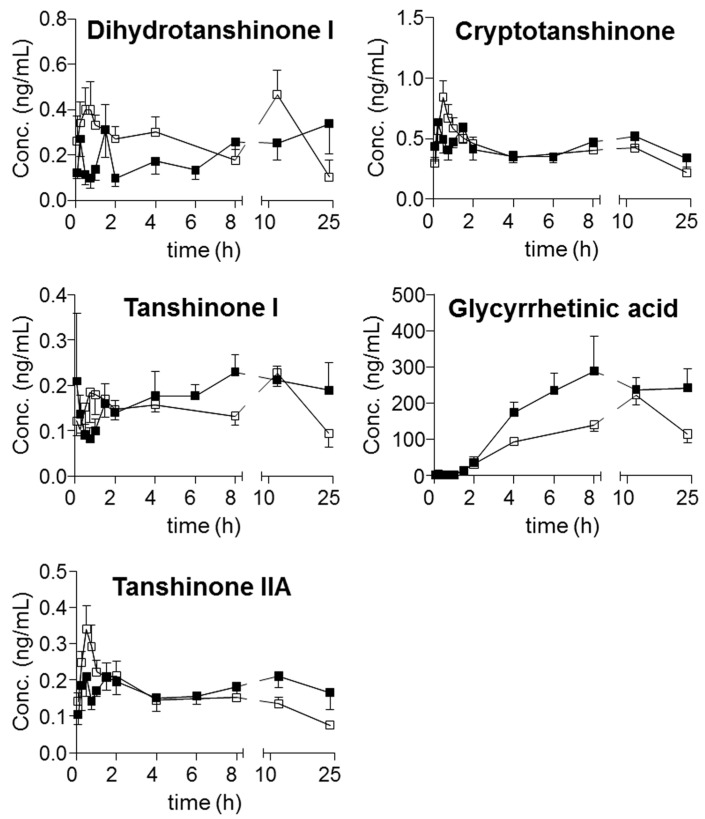
Mean (±SD, *n* = 6) concentration-time profiles of twenty-one analytes in normal (hollow) and model (solid) rat plasma following oral administration of QSKL.

Moreover, harpagoside was always quantifiable in the plasma of the model group, whereas most of the concentrations in the samples from the sham group were lower than its LOQ. Following the achievement of simultaneous determination of the twenty-one compounds in plasma, their trajectories in all QSKL-administrated rats were obtained. Mean plasma concentration-time profiles of the twenty-one analytes are illustrated in Figure 3, and the main pharmacokinetic parameters that were calculated using non-compartmental model are summarized in Table 7. Overall, most compounds reached their maximum concentrations within 2 h, whereas slow absorption was observed for the four diterpenequinonederivatives (T_max_, 8 h). e.g., dihydrotanshinone I, cryptotanshinone, tanshinone I, and tanshinone IIA, as well as glycyrrhetic acid (T_max_ greater than 12 h). Except glycyrrhizic acid and glycyrrhetic acid, relatively rapid elimination (*t*_1/2_ less than 24 h) occurred for the other components after oral dosing QSKL. Multiple-peak profiles occurred for liquiritigenin, isoliquiritigenin, glycyrrhetinic acid, and glycyrrhizic acid.

## 3. Discussion

The pharmacokinetic profiles of QSKL were compared between the sham and model groups. C_max_ of harpagoside in model rats (1.02 ± 0.692 ng/mL) was quite greater than that of sham rats (Table 7), and significant differences were observed for AUC_0→t_ of glycyrrhizic acid (sham: 37.9 ± 7.11 vs. model: 72.8 ± 27.8, ng·h/mL), glycyrrhetic acid (sham: 3205 ± 298 vs. model: 5038 ± 1857, ng·h/mL), and tanshinone IIA (sham: 3.18 ± 0.664 vs. model: 4.28 ± 0.678, ng·h/mL) (Table 7).

Multiple peak phenomena have been widely reported for a number oforal drugs, and several mechanisms have been proposed for the phenomenon, such as enterohepatic recycling, and the presence of absorption windows along the gastrointestinal tract. Moreover, the complicated chemical correlations among the ingredients in TCMs can also contribute to this phenomenon, for instance the generation of aglycones viahydrolysis of the glycosides. In current study, multiple peak profiles were observed for liquiritigenin along with isoliquiritigenin, attributing to the hydrolysis of their glycoside. On the other side, the unique pharmacokinetic curves of glycyrrhetinic acid and glycyrrhizic acid might be attributed to the mutual transformation between these compounds which was governed by enterohepatic recycling [30,31,32,33], hydrolysis of glycyrrhizic acid, and glucuronidation of glycyrrhetinic acid.

Disorders usually result in changes of enzyme expressions and various organs, for instance the modification of gut flora, the damage of intestinal mucosa; hence, the absorption, distribution, metabolism, and excretion (ADME) profiles of those absorbable components might change, due to the changes of the interactions between the components and relevant proteins. In other words, the differences of pharmacokinetic profiles might provide meaningful clues for searching effective components. Moreover, significant exposureshould be afforded by a given component in plasma if it exhibits activity in vivo. Therefore, the pharmacokinetic differences of components, especially those primary ones, between normal and disease animals could provide meaningful information for seeking the effective components. Although the greatest content was observed for sweroside in QSKL extract, more abundant occurrencewas detected for glycyrrhetic acid (C_max_: 230 ± 57.1 ng/mL for sham group and 376 ± 196 ng/mL for model group) than sweroside (C_max_: 28.9 ± 14.3 ng/mL for sham group and 54.2 ± 38.9 ng/mL for model group). In addition to sweroside along with glycyrrhetic acid, the exposure of glycyrrhizic acid (AUC_0→t_: 37.9 ± 7.11 ng·h/mL in sham group and 72.8 ± 27.8, ng·h/mL in model group) was also quite abundant. Moreover, different from sweroside, significant differences occurred between the kinetic profiles, in particular AUC_0→t_ values of glycyrrhizic acid as well as glycyrrhetic acid between the sham and model groups. In addition, significant differences also occurred for C_max_ of harpagoside as well as AUC_0→t_ of tanshinone IIA; however, relative low contents were observed for these two compounds. As a consequence, glycyrrhizic acid together with glycyrrhetic acid might play primary roles in response to myocardial ischemia induced by LAD coronary artery ligation in rats. Actually, the efficacy of these two compounds has been well-defined on cardiac performance [34,35], and the modification of the interactions between these two compounds and their targets should play key roles for those significant differences.

## 4. Materials and Methods 

### 4.1. Materials and Reagents

Authentic compounds, including aconitine, hypaconitine, benzoylmesaconine, benzoyl-hypaconine, dihydrotanshinone I, harpagide, isoacteoside, harpagoside, morroniside, sweroside, glycyrrhizic acid, liquiritigenin, isoliquiritigenin, liquiritin, isoliquiritin, liquiritin apioside, licochalcone A, formononetin, calycosin, luteolin, and luteoloside, were purchased from Shanghai Standard Biotech Co. Ltd. (Shanghai, China), while tanshinone IIA, cryptotanshinone, and tanshinone I, as well as glycyrrhetic acid, were obtained from the National Institutes for Food and Drug Control (Beijing, China). All chemical structures (Figure 1), 25 in total, were further verified by ^1^H-, ^13^C-NMR, and MS analyses, and the purity of each authentic compound was determined to be greater than 98% by normalization of the peak areas detected by UHPLC-IT-TOF-MS (Shimadzu, Kyoto, Japan). Two additional compounds such as mangiferin (IS1) [36] and 1,7-dimethoxyxanthone (IS2) [37] which were previously purified from *Polygala tenuifolia Willd* in our laboratory served as internal standards for positive and negative polarities, respectively.

Formic acid, dimethylsulfoxide (DMSO), and acetonitrile (ACN) were of LC-MS grade and purchased from Merck (Darmstadt, Germany). Ultrapure water was prepared in-house with a Milli-Q system (Millipore, Bedford, MA, USA). The other chemicals were of analytical grade and obtained commercially from Beijing Chemical Works (Beijing, China). Freeze-dried QSKL extract powders were prepared in our group following the previous protocol [15] and the contents of those twenty-five compounds including harpagide (2.7%), morroniside (5.3%), sweroside (17.6%), liquiritin apioside (5.3%), liquiritin (9.2%), luteoloside (0.7%), isoacteoside (0.09%), benzoylmesaconine (4.8%), isoliquiritin (1.7%), benzoylhypaconine (0.7%), harpagoside (2.1%), liquiritigenin (0.9%), luteolin (0.02%), calycosin (0.7%), aconitine (0.07%), hypaconitine (0.2%), isoliquiritigenin (0.4%), glycyrrhizic acid (14.2%), formononetin (0.8%), licochalcone A (0.009%), dihydrotanshinone I (0.2%), cryptotanshinone (0.2%), tanshinone I (0.2%), glycyrrhetic acid (9.7%), and tanshinone IIA (0.4%) were quantified using UHPLC-MS/MS.

### 4.2. Method Development 

An LC-20AD LC system (Shimadzu, Kyoto, Japan) consisting of two LC-20AD_XR_ solvent delivery units, a SIL-20AC_XR_ auto-sampler, a CTO-20AC column oven, a DGU-20-A_3R_ degasser, and a CBM-20A controller, was directly connected with an ABSciex 5500 Q-trap mass spectrometer (Foster City, CA, USA) via an electrospray ionization (ESI) interface. Chromatographic separations were conducted on theACQUITY UPLC HSS T3 column. The mobile phase was composed of 0.02% aqueous formic acid (*v*/*v*, A) and ACN containing 0.02% formic acid (*v*/*v*, B), the gradient elution was programmed as follows: 0–10 min, 5–50% B; 10–15 min, 50–70% B; 15–17 min, 70–95% B; 17–20 min, 95% B; and flow rate, 0.4 mL/min. Re-equilibration of the entire system was achieved by delivering 5% B for 5 min after each program. Column oven was maintained at 35 °C, and the injection volume was set at 10 μL. The auto-sampler chamber was maintained at 4°C.

Regarding the mass spectrometer domain, mass spectrometric detection was operated in multiple reaction monitoring (MRM) mode with programmed polarity switching (Figure 2). Nitrogen acted as the nebulizer (GS1), heater (GS2), and curtain (CUR), as well as collision gas. MS parameters were set as follows: GS1, 55 psi; GS2, 55 psi; CUR, 35 psi; ion spray needle voltage, 5500 V/−4500 V (5500 V and −4500 V for positive and negative polarities, respectively); heater gas temperature, 550 °C; entrance potential (EP), 10 V/−10 V; collision cell exit potential (CXP), 13 V/−16 V. The MRM ion transitions, declustering potential (DP) levels, and collision energy (CE) values, as well as the polarity-switching schedule, are summarized in Table 1. Moreover, MRM mode also acted as survey experiment to trigger two separate enhanced product ion (EPI) scans via information dependent acquisition (IDA)algorithm with a threshold criterion as 200 cps. Key parameters for EPI experiments, such as CE and collision energy spread (CES), were set as ±30 eV and 20 eV, respectively.

### 4.3. Method Validation

The developed method was validated in terms of selectivity, linearity, precision, and accuracy following the FDA Guidance [38].

#### 4.3.1. Linearity and Sensitivity

Based on our preliminary experiments, stock solutions of all authentic compounds were separately prepared with DMSO to appropriate concentrations. Mixed standard stock solution was obtained by pooling all stock solutions of authentic compounds. The solutions were stored at −20 °C until usage.

The mixed stock solution was sequentially diluted with DMSO to yield a series of solutions with desired concentration levels, and a 2 μL aliquot of each one was added into 98 μL of pooled rat plasma that was obtained from six drug-free male Spargue-Dawley rats to generate a set of calibration samples. After thoroughly mixing, protein precipitation was accomplished for each calibration sample by adding three volumes of ACN containing both internal standards (200 ng/mL for either). The precipitates were excluded through 12,000 rpm centrifugation for 10 min at 4 °C. A 300 μL aliquot of the supernatant was transferred into another Eppendorf tube and then concentrated to dryness with nitrogen blow at 40 °C. The residues were reconstituted with 100 μL of 50% aqueous ACN (*v*/*v*) and centrifugation at 12,000 rpm for 10 min was performed to remove the precipitates. Finally, a 10 μL aliquot of the supernatant was injected into UHPLC-*ps*MS/MS system. Each calibration sample was assayed in triplicate. 

To gain acceptable deviations for all concentration levels, the standard curves were fitted by a 1/*x* weighed least squares linear regression method through plotting the peak area ratios of each analyte to respective IS versus the theoretical plasma concentrations over the calibration concentration range. The acceptance criterion for each calibration curve was a correlation coefficient (*r*) of 0.99 or better and a back-calculated standard concentration within a 15% deviation from the nominal value except at the lower limit of quantification (LOQ). The term of LOQ of each analyte was defined as the lowest concentration in the calibration curve with inter-day imprecision less than 20% as well as inter-day inaccuracy less than ±20%, whereas the limit of detection (LOD) of each analyte corresponded to the lowest detective concentration with signal-to-noise ratio (S/N) of about 3. Moreover, quality control (QC) samples with three concentration levels (high, medium, and low) were prepared based on the linear ranges of all analytes.

#### 4.3.2. Selectivity and Specificity

Selectivity and specificity assays were carried out to check the potential chromatographic interferences from endogenous substances around the signals of the analytes as well as the internal standards. Chromatographic peaks from plasma samples were compared with the authentic standards in terms of the retention times and MS^2^ spectra obtained by EPI scans.

#### 4.3.3. Accuracy and Precision

Accuracy and intra- and inter-day precisions were assessed by analyzing three consecutive batches of calibration samples and six replicates of each QC level (low, medium or high concentration level), respectively. The accuracy was expressed as ratio earning (RE, %) which was calculated according to (observed concentration/nominal concentration) × 100% and acceptable at the case of RE within 100 ± 15%, while the relative standard deviations (RSDs, %) within ±15% was acceptable for precision assays.

#### 4.3.4. Matrix Effect and Recovery Assays

Process efficiency (overall method recovery), recovery (extraction yield), and matrix effect were assessed by combined experiments following the descriptions archived in the literature [39]. In brief, independent experiments using pooled drug-free rat plasma along with three diluted mixed standard solutions, corresponding to those QC samples, were performed on three different days with threefold analysis repetitions at each day. The process efficiency (overall method recovery) was determined by comparing the analyte signals (peak areas) obtained from plasma samples spiked prior to extraction (set C) with signals from spiked sample preparation solution (set A). The recovery was determined by comparing analyte responses obtained from set C with signals obtained from plasma samples spiked after the extraction process (set B). Moreover, the matrix effect was evaluated by comparing the analyte peak areas obtained from set A with the ones from set B. The process efficiency (%) is calculated as C/A×100, while the recovery (%) as C/B×100 and the matrix effect (%) as B/A×100, respectively.

#### 4.3.5. Stability Assays

Stability was assayed according to maintaining those QC samples in different conditions. The short-term stability was assessed within the exposure of the QC samples at room temperature for 24 h and measurements were carried out every three hours. The freeze-thaw stability was conducted by evaluating the effects from three freeze-thaw cycles from −80 °C to room temperature (22 °C). The long-term stability was assessed by maintaining QC samples at −80 °C for 30 days.

### 4.4. Pharmacokinetic Study

Twelve male Spargue-Dawley rats (200 ± 20 g) were supplied by Vital River Laboratory Animal Technology Co. Ltd. (Beijing, China). The study protocol was approved by the Committee on the Ethics of Animal Experiments of Beijing University of Chinese Medicine (IACUC approval number 2010-D-013). The rats were acclimated under a humidity (50%) controlled room at 22–25 °C with a 12 h light-dark cycle, and standard diet and water were provided ad libitum for one week. 

Afterwards, all rats were randomly divided into two groups, namely sham and model groups. The myocardial ischemia model was simulated by left anterior descending (LAD) coronary artery ligation [12], whilst the rats belonging to the sham group received the similar surgery, however no actual ligation in LAD. All animals were fasted overnight with free access to water prior to oral dosing. Aliquots (250 μL) of blood were sampled at 0 (pre-dose), 0.08, 0.25, 0.5, 0.75, 1, 1.5, 2, 4, 6, 8, 12, and 24 h from venous around eye socket into heparinized tubes after a single oral dosing of 8.0 g/kg of QSKL and the collected blood samples were immediately subjected for 4000 rpm centrifugation for 10 min at 4 °C to harvest the plasma samples. Each 100 μL aliquot of plasma sample was transferred into another Eppendorf tube and stored at −80 °C till use. 

When analysis, 300 μL of internal standards (200 ng/mL for either internal standard) fortified ACN, corresponding to three volumes of plasma, was spiked into each thawed plasma sample and mixed thoroughly. Then, the resulting samples were processed as those calibration samples. 

Individual animal plasma concentrations versus time dataset were subjected to a non-compartmental pharmacokinetic analysis using WinNonlin software (ver. 6.4, Pharsight Corp., Sunnyvale, CA, USA). The pharmacokinetic parameters of each analyte including the maximum plasma concentration (C_max_), the time at which C_max_ was achieved (T_max_), the terminal elimination half-life (*t*_1/2_), and the area under the plasma concentration versus time curve (AUC_0→t_ along with AUC_0→∞_) were calculated. Moreover, Lz corresponding to the ratio of the dosage and AUC_0→∞_, as well as mean residence time (MRT) that was defined as the ratio of the area under the first moment curve from time 0 to infinity (AUMC_0→∞_/AUC_0→∞_) was also calculated. All results are expressed as mean ± SD. Comparisons regarding the pharmacokinetic parameters were carried out between sham and model groups using Student’s *t*-test, and *p* values less than 0.05 were considered to be significant.

## 5. Conclusions

In the present study, a reliable and sensitive method using UHPLC-*ps*MS/MS was developed and validated for the simultaneous determination of as many as twenty-five compounds in rat plasma. Scheduled polarity switching was programed for the ion source of the mass spectrometer to meet the demands of simultaneous monitoring both positive ionization-and negative ionization-favored compounds, while a robust ACQUITY UPLC HSS T3 column afforded satisfactory chromatographic performance in terms of measurement time, peak shape and resolution. Formic acid was found to be suitable for improving the peak shapes, and thereby used as the additive in the mobile phase. A variety of method validation assays demonstrated the method to be specific, sensitive, and accurate. Except those four unquantifiable components (isoacteoside, luteolin, aconitine, and licochalcone A) as well as harpagoside, the pharmacokinetic profiles of the other twenty analytes in both sham and model rats were acquired by applying the validated method for QSKL-treated plasma samples. Significant different pharmacokinetic patternswere observed for glycyrrhizic acid and glycyrrhetic acid between the sham and model rats, whereas mild differences occurred for the other analytes. Hence, the contributions of these twenty-five components, in particular glycyrrhizic acid and glycyrrhetic acid, for the therapeutic outcomes of QSKL can be plausibly claimed. Overall, the information obtained from this study might provide some meaningful clues and evidences for the further studies regarding the clarification of the effective material basis of QSKL and is alsobeneficial for its clinical applications.

## Figures and Tables

**Table 1:** The retention times (*t*_R_), MRM ion transitions, declustering potentials (DPs), collision energies (CEs) of the targeted components and twelve periods of the polarity switching mass parameters.

Period	Duration (min)	Analyte	*t*_R_ (min)	Ion Transition Precursor > Product Ion ^1^	DP (V)	CE (eV)
1 (Neg.)	0–6.15	Harpagide	2.80	**409 > 363**; 363 > 201	–100	–15
		Morroniside	3.51	**405 > 243**; 405 > 155	–70	–13
		Mangiferin (IS1)	4.12	**421 > 331**; 421 > 301	–140	–31
		Sweroside	4.48	**403 > 357**; 357 > 195	–100	–15
		Liquiritin apioside	5.21	549 > 417; **549 > 255**	–150	–42
		Liquiritin	5.30	**417 > 255**; 417 > 135	–130	–26
		Luteoloside	5.56	**447 > 285**; 447 > 133	–160	–36
		Isoacteoside	5.73	**623 > 161**; 623 > 461	–120	–44
2 (Pos.)	6.15–6.51	Benzoylmesaconine	6.35	590 > 558; **590 > 540**	90	48
3 (Neg.)	6.51–6.81	Isoliquiritin	6.68	**417 > 255**; 417 > 135	–160	–25
4 (Pos.)	6.81–7.11	Benzoylhypaconine	7.14	**574 > 542**; 574 > 510	103	47
5 (Neg.)	7.11–7.81	Harpagoside	7.26	**539 > 493**; 493 > 345	–100	–15
		Liquiritigenin	7.27	255 > 135; **255 > 119**	–120	–30
		Luteolin	7.38	285 > 175; **285 > 133**	–150	–40
		Calycosin	7.57	**283 > 268**; 283 > 239	–100	–25
6 (Pos.)	7.81–8.61	Aconitine	8.46	**646 > 586**; 646 > 526	120	44
		Hypaconitine	8.56	**616 > 556**; 616 > 524	130	44
7 (Neg.)	8.61–10.01	Isoliquiritigenin	9.57	255 > 135; **255 > 119**	–90	–30
		Glycyrrhizic acid	9.67	821 > 645; **821 > 351**	–40	–55
		Formononetin	9.84	**267 > 252**; 267 > 223	–130	–28
8 (Pos.)	10.01–10.61	1,7-dimethoxyxanthone (IS2)	10.30	**257 > 242**; 257 > 227	120	30
9 (Neg.)	10.61–12.22	Licochalcone A	11.80	337 > 305; **337 > 187**	–100	–46
10 (Pos.)	12.22–15.02	Dihydrotanshinone I	12.62	**279 > 223**; 279 > 205	90	30
		Cryptotanshinone	14.47	297 > 279; **297 > 251**	60	34
		Tanshinone I	14.54	**277 > 249**; 277 > 193	150	28
11 (Neg.)	15.02–16.32	Glycyrrhetic acid	16.14	**469 > 425**; 469 > 355	–20	–51
12 (Pos.)	16.32–20.00	Tanshinone IIA	16.64	**295 > 277**; 295 > 249	180	27

^1^: Two ion transitions were optimized for each analyte, and the ion transitions in bold were implemented for quantitative analysis.

**Table 2:** Regression equations, linear ranges, LOQs, and LODs of the twenty-five analytes.

Analyte	Linear Regression Data			LOQ(ng/mL)	LOD(ng/mL)
	Regression Equation	*r*	Linear Range (ng/mL)		
Harpagide	y = 0.085x + 0.025	0.9992	0.192–197	0.192	0.0913
Morroniside	y = 0.00785x− 0.00321	0.9990	0.534–2190	0.534	0.267
Sweroside	y = 0.00527x + 0.0139	0.9994	3.78–1930	3.78	1.89
Liquiritin apioside	y = 0.364x + 0.00801	0.9991	0.0146–29.7	0.0146	0.00730
Liquiritin	y = 0.714x + 0.156	0.9995	0.0882–22.6	0.0882	0.0110
Luteoloside	y = 0.634x− 0.00473	0.9994	0.0237–12.1	0.0237	0.0118
Isoacteoside	y = 0.00108x + 0.0968	0.9983	6.60–3370	6.60	1.65
Benzoylmesaconine	y = 0.145x + 0.0478	0.9978	0.0621–31.8	0.0621	0.00190
Isoliquiritin	y = 0.451x + 0.00118	0.9990	0.0111–22.6	0.0111	0.00553
Benzoylhypaconine	y = 0.441x + 0.0189	0.9990	0.0151–30.9	0.0151	0.00190
Harpagoside	y = 0.0877x + 0.00215	0.9990	0.0665–267	0.0665	0.0333
Liquiritigenin	y = 0.776x + 0.00849	0.9991	0.0135–13.8	0.0135	0.00670
Luteolin	y = 0.498x − 0.008	0.9991	0.121–15.4	0.121	0.0600
Calycosin	y = 1.41x− 0.0047	0.9994	0.0153–15.3	0.0153	0.00750
Aconitine	y = 0.338x− 0.045	0.9990	0.272–34.8	0.272	0.100
Hypaconitine	y = 0.301x + 0.0197	0.9991	0.0808–33.2	0.0808	0.00300
Isoliquiritigenin	y = 1.57x + 0.00961	0.9992	0.0135–13.8	0.0135	0.000900
Glycyrrhizic acid	y = 0.00763x + 0.026	0.9991	0.217–444	0.217	0.0272
Formononetin	y = 2.72x + 0.0772	0.9993	0.00711–14.5	0.00711	0.00350
Licochalcone A	y = 0.262x + 0.0221	0.9993	0.0713–9.13	0.0713	0.0356
Dihydrotanshinone I	y = 0.066x + 0.021	0.9987	0.0735–37.5	0.735	0.0367
Cryptotanshinone	y = 0.0212x + 0.0161	0.9992	0.156–160	0.156	0.00980
Tanshinone I	y = 0.113x + 0.00791	0.9990	0.0728–74.5	0.0728	0.0364
Glycyrrhetic acid	y = 0.0483x− 0.000447	0.9990	1.24–2540	1.24	0.310
Tanshinone IIA	y = 0.185x + 0.017	0.9992	0.0390–159	0.0390	0.0190

**Table 3:** Precision and accuracy data for the twenty-five ananlytes (*n* = 6).

Analyte	Concentration (ng/mL)	Accuracy RE (%)	Intra-Day RSD (%)	Inter-Day RSD (%)	Analyte	Concentration (ng/mL)	Accuracy RE (%)	Intra-Day RSD (%)	Inter-Day RSD (%)
Harpagide	0.384	93.4	13.6	8.1	Calycosin	0.0306	95.1	3.8	4.0
	6.14	95.8	12.8	14.9		0.490	98.4	10.7	10.1
	98.3	101.0	8.55	9.8		7.83	100.0	8.8	8.4
Morroniside	2.14	90.8	9.7	8.4	Aconitine	0.544	115.0	5.0	7.2
	34.2	94.9	7.4	6.1		4.35	110.0	3.1	6.0
	548	98.7	9.9	10.2		17.4	108.0	7.8	10.6
Sweroside	7.56	88.4	8.4	11.6	Hypaconitine	0.0324	114.0	6.2	5.9
	121	92.3	7.8	9.9		0.518	111.0	2.9	6.0
	968	105.0	14.0	14.6		16.6	110.0	3.8	5.8
Liquiritin apioside	0.0292	110.0	13.9	9.4	Isoliquiritigenin	0.0270	96.4	6.2	4.8
	0.467	103.0	14.4	11.3		0.432	98.1	5.8	8.0
	14.9	98.9	10.7	13.9		6.91	100.0	8.0	6.8
Liquiritin	0.176	112.0	13.7	13.0	Glycyrrhizic acid	0.434	95.7	14.5	11.6
	1.41	108.0	9.4	9.9		6.94	96.9	11.9	14.1
	11.3	103.0	11.0	11.4		222	101.0	11.8	13.4
Luteoloside	0.0474	90.4	8.7	7.8	Formononetin	0.0284	92.4	7.8	7.7
	0.758	93.5	8.5	12.5		0.454	95.1	8.4	9.1
	6.07	97.6	7.4	9.0		7.27	102.0	6.8	8.3
Isoacteoside	13.2	87.5	12.2	14.1	Licochalcone A	0.143	108.0	8.9	6.0
	211	93.1	9.0	13.6		1.14	103.0	7.4	5.9
	1690	101.0	13.0	13.1		4.56	98.9	6.7	7.8
Benzoylmesaconine	0.124	85.3	2.4	3.4	Dihydrotanshinone I	0.294	93.1	15.5	12.2
	1.99	88.9	2.8	3.0		2.35	95.8	13.4	13.8
	15.9	105.0	3.3	5.6		18.8	97.3	13.3	15.5
Isoliquiritin	0.022	90.4	5.3	4.5	Cryptotanshinone	0.312	91.2	6.8	8.9
	0.355	95.1	9.4	12.8		4.99	93.8	5.5	14.9
	11.4	98.8	6.5	7.0		79.9	97.0	8.3	14.2
Benzoylhypaconine	0.0606	88.1	3.4	2.8	Tanshinone I	0.146	93.1	6.3	5.2
	0.969	94.2	1.9	3.8		2.33	95.6	4.0	5.9
	15.5	99.3	3.3	6.2		37.3	97.1	4.6	7.9
Harpagoside	0.522	105.0	13.0	10.4	Glycyrrhetic acid	2.48	105.0	7.2	3.6
	8.35	102.0	14.5	10.7		39.7	103.0	6.8	9.5
	134	98.7	12.6	6.1		1270	99.8	9.3	8.9
Liquiritigenin	0.0270	106.0	7.2	7.1	Tanshinone IIA	0.156	94.9	2.9	3.9
	0.432	104.0	8.1	5.8		2.50	96.1	3.3	6.1
	6.91	99.5	6.8	9.4		80.0	98.9	2.9	8.2
Luteolin	0.242	107.0	9.1	7.4					
	1.94	104.0	6.4	15.0					
	7.74	100.0	9.1	8.9					

**Table 4:** Recovery data for the twenty-five analytes (*n* = 6).

Analyte	Concentration (ng/mL)	Recovery (%)	RSD (%)	Analyte	Concentration(ng/mL)	Recovery (%)	RSD (%)
Harpagide	0.384	62.7	13.7	Calycosin	0.0306	90.0	11.7
	6.14	67.2	10.3		0.490	83.1	8.3
	98.3	71.8	8.7		7.83	76.4	6.5
Morroniside	2.14	90.0	8.9	Aconitine	0.544	81.4	10.9
	34.2	86.3	7.5		4.35	79.8	5.9
	548	80.7	2.7		17.4	76.6	1.7
Sweroside	7.56	88.9	8.9	Hypaconitine	0.0324	89.9	9.9
	121	82.3	8.7		0.518	84.3	8.4
	968	77.8	5.5		16.6	76.1	4.2
Liquiritin apioside	0.0292	61.3	13.8	Isoliquiritigenin	0.0270	61.8	11.1
	0.467	64.3	11.6		0.432	65.7	10.7
	14.9	70.4	10.7		6.91	70.3	6.5
Liquiritin	0.176	63.1	13.2	Glycyrrhizic acid	0.434	68.5	7.4
	1.41	69.1	10.9		6.94	71.9	5.2
	11.3	72.0	8.9		222	73.0	3.9
Luteoloside	0.0474	61.5	13.9	Formononetin	0.0284	90.2	12.9
	0.758	66.6	11.4		0.454	84.1	11.8
	6.07	70.2	10.3		7.27	77.9	6.5
Isoacteoside	13.2	71.3	8.9	Licochalcone A	0.143	66.4	12.7
	211	78.1	6.4		1.14	69.8	9.0
	1690	74.8	4.0		4.56	74.3	6.3
Benzoylmesaconine	0.124	69.3	11.7	Dihydrotanshinone I	0.294	85.3	11.7
	1.99	70.2	10.9		2.35	82.1	8.7
	15.9	72.1	9.9		18.8	80.0	6.4
Isoliquiritin	0.022	62.4	12.9	Cryptotanshinone	0.312	84.1	12.0
	0.355	69.1	10.7		4.99	81.3	8.9
	11.4	72.4	6.3		79.9	78.9	6.1
Benzoylhypaconine	0.0606	66.5	12.8	Tanshinone I	0.146	87.9	10.8
	0.969	77.1	11.0		2.33	83.4	5.2
	15.5	76.9	10.0		37.3	80.8	3.8
Harpagoside	0.522	70.4	9.8	Glycyrrhetic acid	2.48	69.3	8.9
	8.35	78.8	7.7		39.7	71.1	5.7
	134	74.6	5.3		1270	73.2	1.3
Liquiritigenin	0.0270	90.4	11.5	Tanshinone IIA	0.156	80.3	10.7
	0.432	85.1	6.4		2.50	78.4	8.5
	6.91	70.4	3.1		80.0	76.6	5.3
Luteolin	0.242	87.2	10.7				
	1.94	81.1	4.2				
	7.74	70.4	3.6				

**Table 5:** Matrix effect data for the twenty-five analytes (*n* = 6).

Analyte	Concentration (ng/mL)	Accuracy (%)	RSD (%)	Analyte	Concentration(ng/mL)	Accuracy (%)	RSD (%)
Harpagide	0.384	95.7	8.9	Calycosin	0.0306	105.0	2.5
	6.14	90.6	9.2		0.490	96.1	8.4
	98.3	92.8	9.7		7.83	99.4	5.6
Morroniside	2.14	95.2	10.1	Aconitine	0.544	99.4	7.9
	34.2	96.6	7.2		4.35	105.8	5.7
	548	104.7	1.0		17.4	101.6	1.8
Sweroside	7.56	98.9	8.9	Hypaconitine	0.0324	103.9	9.1
	121	93.3	8.7		0.518	95.3	8.4
	968	95.8	5.5		16.6	92.1	4.5
Liquiritin apioside	0.0292	98.3	8.8	Isoliquiritigenin	0.0270	94.8	7.2
	0.467	94.5	7.6		0.432	98.7	9.7
	14.9	100.6	9.7		6.91	97.7	6.0
Liquiritin	0.176	97.1	9.2	Glycyrrhizic acid	0.434	93.5	3.3
	1.41	102.8	8.9		6.94	90.9	6.6
	11.3	92.0	8.9		222	100.2	1.8
Luteoloside	0.0474	91.5	6.9	Formononetin	0.0284	97.2	2.3
	0.758	105.6	8.4		0.454	100.3	1.9
	6.07	95.2	4.3		7.27	105.9	6.6
Isoacteoside	13.2	100.3	8.9	Licochalcone A	0.143	98.6	2.0
	211	102.1	6.4		1.14	104.7	9.3
	1690	95.8	3.0		4.56	102.3	6.6
Benzoylmesaconine	0.124	105.3	5.7	Dihydrotanshinone I	0.294	92.1	1.6
	1.99	91.6	7.9		2.35	101.2	8.3
	15.9	99.1	2.9		18.8	107.0	6.7
Isoliquiritin	0.022	95.4	5.9	Cryptotanshinone	0.312	98.2	2.0
	0.355	92.1	4.7		4.99	97.3	8.3
	11.4	98.4	8.3		79.9	101.8	6.5
Benzoylhypaconine	0.0606	105.5	7.8	Tanshinone I	0.146	97.7	3.8
	0.969	97.1	6.0		2.33	95.4	5.9
	15.5	97.8	8.0		37.3	100.9	3.8
Harpagoside	0.522	95.4	9.8	Glycyrrhetic acid	2.48	105.5	8.5
	8.35	102.8	5.7		39.7	92.6	5.7
	134	107.6	4.3		1270	97.2	1.4
Liquiritigenin	0.0270	105.4	2.5	Tanshinone IIA	0.156	100.7	8.8
	0.432	105.1	5.4		2.50	97.4	8.9
	6.91	107.4	6.1		80.0	96.9	5.3
Luteolin	0.242	99.2	10.0				
	1.94	96.1	5.2				
	7.74	100.4	3.7				

**Table 6:** Stability data for the twenty-five analytes (*n* = 6).

Analyte	Concentration Level (ng/mL)	22 °C for 24 h		Frozen for 30 Days		Three-Freeze-Thaw Cycles	
		AccuracyRE (%)	RSD (%)	AccuracyRE (%)	RSD (%)	AccuracyRE (%)	RSD (%)
Harpagide	0.384	115.0	15.3	88.5	15.8	89.0	13.8
	6.14	105.0	11.7	90.0	10.5	110.0	9.1
	98.3	99.8	4.9	107.0	9.8	91.5	10.6
Morroniside	2.14	94.1	10.2	87.4	14.1	89.5	12.3
	34.2	106.0	9.3	113.0	4.3	108.0	5.8
	548	95.5	6.4	96.5	10.3	102.0	5.8
Sweroside	7.56	93.0	12.5	119.0	6.8	101.0	13.2
	121	107.0	9.9	94.6	2.2	101.0	9.5
	968	96.4	3.3	95.9	5.4	107.0	6.1
Liquiritin apioside	0.0292	111.0	15.9	115.0	13.1	108.0	15.0
	0.467	110.0	12.7	110.0	12.6	98.5	5.9
	14.9	92.2	7.76	97.2	10.5	99.7	6.1
Liquiritin	0.176	114.0	4.6	114.0	14.3	112.0	8.3
	1.41	110.0	3.2	108.0	10.9	96.1	6.7
	11.3	97.6	2.7	104.0	9.8	101.0	2.1
Luteoloside	0.0474	117.0	13.8	114.0	11.8	106.0	15.8
	0.758	109.0	10.2	109.0	10.5	103.0	4.1
	6.07	107.0	7.8	105.0	8.8	107.0	3.2
Isoacteoside	13.2	108.0	8.9	110.0	11.3	113.0	14.3
	211	104.0	7.6	109.0	8.9	109.0	12.8
	1690	98.4	4.2	103.0	7.4	108.0	8.4
Benzoylmesaconine	0.124	113.0	6.0	113.0	11.2	84.8	13.9
	1.99	112.0	3.7	108.0	10.9	90.7	10.7
	15.9	105.0	2.8	105.0	1.9	94.1	8.4
Isoliquiritin	0.022	115.0	13.9	109.0	10.7	84.8	12.7
	0.355	110.0	10.7	106.0	9.5	88.7	10.4
	11.4	108.0	5.2	98.6	6.3	94.7	3.7
Benzoylhypaconine	0.0606	115.0	6.6	112.0	9.4	114.0	10.1
	0.969	112.0	2.1	112.0	6.3	110.0	8.5
	15.5	108.0	3.5	110.0	2.5	92.7	4.2
Harpagoside	0.522	113.0	12.0	113.0	12.9	115.0	11.1
	8.35	104.0	10.4	110.0	9.4	86.9	10.8
	134	97.0	7.7	98.4	7.3	104.0	9.2
Liquiritigenin	0.0270	115.0	11.9	117	10.7	110.0	12.4
	0.432	109.0	8.8	113	8.9	92.1	10.4
	6.91	106.0	6.5	108	6.4	98.9	8.5
Luteolin	0.242	111.0	10.9	112	12.4	84.5	13.6
	1.94	108.0	9.3	106	11.0	89.1	8.8
	7.74	98.7	9.3	103	8.5	96.9	8.9
Calycosin	0.0306	114.0	6.0	93.8	9.0	109.0	14.7
	0.490	113.0	5.9	111.0	9.0	99.8	9.9
	7.83	108.0	4.1	98.4	2.3	101.0	4.1
Aconitine	0.544	115.0	6.3	88.9	6.1	113.0	13.6
	4.35	113.0	5.5	113.0	4.1	108.0	7.8
	17.4	109.0	3.0	110.0	5.1	95.6	2.4
Hypaconitine	0.0324	110.0	11.7	109.0	6.2	84.1	13.2
	0.518	106.0	6.4	103.0	5.9	86.4	9.8
	16.6	104.0	6.2	104.0	3.8	88.4	1.1
Isoliquiritigenin	0.0270	113.0	13.6	110.0	14.5	111.0	6.6
	0.432	98.3	12.4	91.5	11.1	95.1	9.5
	6.91	101.0	10.7	93.6	8.7	100.0	3.2
Glycyrrhizic acid	0.434	109.0	11.8	107.0	8.7	113.0	10.7
	6.94	92.4	9.9	104.0	6.1	110.0	3.6
	222	105.0	8.7	101.0	5.0	98.4	2.2
Formononetin	0.0284	112.0	12.1	114.0	13.9	115.0	8.4
	0.454	108.0	12.3	107.0	10.6	113.0	6.3
	7.27	106.0	8.1	104.0	3.0	109.0	3.2
Licochalcone A	0.143	112.0	14.4	116.0	13.2	91.3	11.3
	1.14	107.0	12.3	114.0	5.8	92.1	8.7
	4.56	108.0	10.6	111.0	0.5	104.0	3.2
Dihydrotanshinone I	0.294	91.1	13.9	115.0	11.4	113.0	6.2
	2.35	96.8	9.9	110.0	6.0	108.0	5.1
	18.8	98.7	6.3	101.0	6.9	96.7	1.1
Cryptotanshinone	0.312	85.7	14.3	87.1	10.3	108.0	11.5
	4.99	88.0	9.0	110.0	8.8	106.0	10.5
	79.9	91.0	6.4	97.1	4.4	105.0	5.8
Tanshinone I	0.146	83.3	10.6	114.0	10.7	88.0	11.5
	2.33	91.4	10.4	91.0	10.6	91.1	9.2
	37.3	95.2	5.4	98.0	3.5	106.0	5.0
Glycyrrhetic acid	2.48	87.3	10.6	85.8	14.2	84.0	11.4
	39.7	107.0	12.5	93.9	10.9	88.9	10.9
	1270	102.0	11.4	105.0	11.3	109.0	9.3
Tanshinone IIA	0.156	85.4	11.4	96.3	6.5	81.6	9.2
	2.50	88.7	13.1	101.0	6.4	86.0	6.5
	80.0	95.3	11.2	101.0	3.7	86.7	6.6

**Table 7:** Pharmacokinetic parameters (mean ± SD) of analytes after oral administration of QSKL in rats (*n* = 6).

Analyte	Group	T_max_ (h)	C_max_ (ng/mL)	AUC_0–t_ (ng·h/mL)	AUC_0–∞_ (ng·h/mL)	Lz (L/h)	*t*_1/2_ (h)	MRT (h)
Harpagide	SG	1.25 ± 1.01	0.590 ± 0.294	2.87 ± 1.16	3.63 ± 1.31	0.0500± 0.0239	8.65 ± 2.06	14.1 ± 3.84
	MG	0.67 ± 0.52	2.83 ± 2.59	6.17 ± 3.32	6.88 ± 3.71	0.151 ± 0.122	6.42 ± 2.99	7.46 ± 3.49
Morroniside	SG	0.37 ± 0.19	2.04 ± 1.07	16.5 ± 3.91	24.9 ± 2.95	0.0540 ± 0.0234	15.8 ± 9.38	25.1 ± 13.5
	MG	0.46 ± 0.25	4.21 ± 3.41	19.5 ± 3.53	29.2 ± 4.10	0.0560 ± 0.0211	14.2 ± 5.79	21.1 ± 8.23
Sweroside	SG	1.58 ± 1.31	28.9 ± 14.3	210 ± 75.7	294 ± 94.2	0.0680 ± 0.0340	16.6 ± 10.6	22.2 ± 20.9
	MG	0.83 ± 0.63	54.2 ± 38.9	247 ± 58.0	281 ± 53.4	0.107 ± 0.0210	6.66 ± 1.09	10.3 ± 2.20
Liquiritin apioside	SG	0.36 ± 0.31	0.800 ± 0.440	2.91 ± 1.11	3.85 ± 1.38	0.104 ± 0.0484	8.54 ± 5.59	12.5 ± 5.12
	MG	0.40 ± 0.23	2.39 ± 1.37	3.59 ± 2.08	4.16 ± 2.41	0.126 ± 0.0700	8.15 ± 5.97	9.22 ± 6.33
Liquiritin	SG	0.29 ± 0.17	1.69 ± 0.78	6.01 ± 1.52	7.37 ± 2.08	0.121 ± 0.00700	5.76 ± 0.360	10.3 ± 2.30
	MG	0.32 ± 0.21	4.62 ± 3.86	7.66 ± 2.60	9.14 ± 4.02	0.114 ± 0.0579	9.46 ± 9.10	10.2 ± 5.77
Luteoloside	SG	0.50 ± 0.33	0.0820 ± 0.0663	0.406 ± 0.055	1.06 ± 0.47	0.0200 ± 0.0103	36.5 ± 16.8	50.5 ± 26.9
	MG	0.35 ± 0.24	0.269 ± 0.264	0.493 ± 0.147	0.869 ± 0.272	0.0440 ± 0.0238	20.3 ± 13.0	29.4 ± 17.5
Benzoylmesaconine	SG	0.42 ± 0.37	1.15 ± 0.778	5.82 ± 2.93	8.30 ± 6.14	0.0650 ± 0.0376	13.0 ± 6.34	20.1 ± 10.3
	MG	0.47 ± 0.33	3.10 ± 2.49	7.34 ± 7.10	11.1 ± 11.0	0.0850 ± 0.0401	9.77 ± 5.36	13.7 ± 6.58
Isoliquiritin	SG	0.36 ± 0.31	0.389 ± 0.167	1.39 ± 0.360	1.64 ± 0.474	0.113 ± 0.0173	6.26 ±0.976	10.5 ± 3.13
	MG	0.43 ± 0.36	0.850 ± 0.761	1.58 ± 0.493	1.86 ± 0.632	0.0970 ± 0.0406	8.28 ± 3.40	10.2 ± 4.65
Benzoylhypaconine	SG	0.48 ± 0.33	0.284 ± 0.209	0.985 ± 0.167	1.78 ± 0.905	0.0530± 0.0390	15.6 ± 9.06	22.7 ± 14.9
	MG	0.43 ± 0.36	0.466 ± 0.302	0.926 ± 0.279	1.66 ± 0.331	0.0460 ± 0.0158	16.3 ± 5.46	23.6 ± 7.39
Harpagoside	SG	-	-	-	-	-	-	-
	MG	0.56 ± 0.38	1.02 ± 0.692 *	2.40 ± 2.04	2.20 ± 1.77	0.0780 ± 0.0519	16.1 ± 11.3	18.7 ± 16.7
Liquiritigenin	SG	4.71 ± 4.18	0.347 ± 0.276	2.11 ± 1.73	2.22 ± 1.98	0.188 ± 0.0467	3.90 ± 1.03	8.48 ± 1.07
	MG	0.69 ± 0.54	0.586 ± 0.271	2.53 ± 0.662	2.81 ± 0.98	0.163 ± 0.107	6.17 ± 3.68	9.83 ± 4.37
Calycosin	SG	0.33 ± 0.29	0.300 ± 0.244	1.03 ± 0.181	3.09 ± 1.95	0.0260 ± 0.0195	38.2 ± 24.5	55.9 ± 34.6
	MG	0.28 ± 0.24	0.347 ± 0.197	0.890 ± 0.284	1.04 ± 0.268	0.0800 ± 0.0275	9.27 ± 3.21	15.0 ± 4.54
Hypaconitine	SG	2.08 ± 1.60	0.101 ± 0.0328	0.824 ± 0.249	0.967 ± 0.303	0.0830 ± 0.0179	8.67 ± 1.72	11.7 ± 2.83
	MG	1.54 ± 0.71	0.156 ± 0.134	0.567 ± 0.260	0.552 ± 0.148	0.0950 ± 0.0165	7.45 ± 1.18	11.9 ± 3.73
Isoliquiritigenin	SG	0.15 ± 0.13	0.389 ± 0.137	1.87 ± 1.21	2.07 ± 1.31	0.170 ± 0.0462	4.37 ± 1.37	7.85 ± 0.600
	MG	0.37 ± 0.28	0.401 ± 0.103	1.56 ± 0.668	1.71 ± 0.740	0.170 ± 0.0659	4.69 ± 2.06	8.14 ± 2.95
Glycyrrhizic acid	SG	1.46 ± 1.35	3.34 ± 1.12	37.9 ± 7.11	130 ± 69.1	0.0230 ± 0.0190	43.2 ± 25.4	63.7 ± 34.4
	MG	0.64 ± 0.50	8.87 ± 5.97	72.8 ± 27.8 *	170 ± 69.1	0.0300 ± 0.0152	27.8 ± 14.2	40.2 ± 21.7
Formononetin	SG	0.61 ± 0.53	0.243 ± 0.127	0.966 ± 0.351	1.22 ± 0.609	0.184 ± 0.0943	4.70 ± 2.32	7.00 ± 2.97
	MG	0.51 ± 0.46	0.470 ± 0.291	1.20 ± 0.482	1.32 ± 0.472	0.226 ± 0.112	3.81 ± 1.89	5.47 ± 2.56
Dihydrotanshinone I	SG	6.38 ± 6.16	0.618 ± 0.250	4.99 ± 2.18	4.47± 0.864	0.182 ± 0.0402	3.90 ± 0.858	8.22 ± 1.20
	MG	9.18 ± 8.77	0.658 ± 0.443	4.67 ± 2.23	5.98 ± 5.34	0.133 ± 0.0927	7.61 ± 5.60	14.8 ± 9.29
Cryptotanshinone	SG	1.88 ± 1.50	0.898 ± 0.290	8.77 ± 2.26	12.0 ± 5.01	0.0630 ± 0.0203	12.2 ± 5.20	19.3 ± 6.01
	MG	8.42 ± 8.39	0.869 ± 0.278	10.2 ± 2.21	16.4 ± 8.86	0.0600 ± 0.0321	17.6 ± 15.6	25.6 ± 20.1
Tanshinone I	SG	12.10 ± 7.35	0.297 ± 0.115	3.62 ± 0.763	-	-	-	-
	MG	8.26 ± 8.14	0.407 ± 0.293	4.54 ± 1.58	5.58 ± 2.56	0.0650 ± 0.00750	10.7 ± 1.20	18.2 ± 2.76
Glycyrrhetic acid	SG	14.00 ± 4.90	230 ± 57.1	3205 ± 298	-	-	-	-
	MG	13.30 ± 8.55	376 ± 196	5038 ± 1857 *	14452 ± 5752	0.0330 ± 0.0176	26.3 ± 15.6	41.9 ± 21.5
Tanshinone IIA	SG	0.67 ± 0.30	0.376 ± 0.150	3.18 ± 0.664	4.77 ± 1.45	0.0550 ± 0.0189	13.8 ± 4.05	20.9 ± 5.14
	MG	8.71 ± 8.03	0.349 ± 0.089	4.28 ± 0.678 *	7.17 ± 3.08	0.0550 ± 0.0409	18.0 ± 10.8	26.4 ± 14.9

SG: the sham group; MG: the model group; * Significant difference between sham and model rats was observed (*p* < 0.05, *t*-test).
